# RNA silencing-related genes contribute to tolerance of infection with potato virus X and Y in a susceptible tomato plant

**DOI:** 10.1186/s12985-020-01414-x

**Published:** 2020-10-08

**Authors:** Joon Kwon, Atsushi Kasai, Tetsuo Maoka, Chikara Masuta, Teruo Sano, Kenji S. Nakahara

**Affiliations:** 1 Graduate School of Agriculture, Hokkaido University, Sapporo, Hokkaido 060-8589 Japan; 2 Faculty of Agriculture and Life Science, Hirosaki University, Hirosaki, 036-8561 Japan; 3 Division of Agro-Environmental Research, Hokkaido Agricultural Research Center, NARO, Sapporo, Hokkaido 062-8555 Japan; 4 Research Faculty of Agriculture, Hokkaido University, Sapporo, Hokkaido 060-8589 Japan

**Keywords:** Dicer-like protein 2, Dicer-like protein 4, Argonaute 2, Argonaute 3, Potato virus X, Potato virus Y

## Abstract

**Background:**

In plants, the RNA silencing system functions as an antiviral defense mechanism following its induction with virus-derived double-stranded RNAs. This occurs through the action of RNA silencing components, including Dicer-like (DCL) nucleases, Argonaute (AGO) proteins, and RNA-dependent RNA polymerases (RDR). Plants encode multiple AGOs, DCLs, and RDRs. The functions of these components have been mainly examined in *Arabidopsis thaliana* and *Nicotiana benthamiana*. In this study, we investigated the roles of DCL2, DCL4, AGO2, AGO3 and RDR6 in tomato responses to viral infection. For this purpose, we used transgenic tomato plants (*Solanum lycopersicum* cv. Moneymaker), in which the expression of these genes were suppressed by double-stranded RNA-mediated RNA silencing.

**Methods:**

We previously created multiple DCL (i.e., DCL2 and DCL4) (hpDCL2.4) and RDR6 (hpRDR6) knockdown transgenic tomato plants and here additionally did multiple AGO (i.e., AGO2 and AGO3) knockdown plants (hpAGO2.3), in which double-stranded RNAs cognate to these genes were expressed to induce RNA silencing to them. Potato virus X (PVX) and Y (PVY) were inoculated onto these transgenic tomato plants, and the reactions of these plants to the viruses were investigated. In addition to observation of symptoms, viral coat protein and genomic RNA were detected by western and northern blotting and reverse transcription-polymerase chain reaction (RT-PCR). Host mRNA levels were investigated by quantitative RT-PCR.

**Results:**

Following inoculation with PVX, hpDCL2.4 plants developed a more severe systemic mosaic with leaf curling compared with the other inoculated plants. Systemic necrosis was also observed in hpAGO2.3 plants. Despite the difference in the severity of symptoms, the accumulation of PVX coat protein (CP) and genomic RNA in the uninoculated upper leaves was not obviously different among hpDCL2.4, hpRDR6, and hpAGO2.3 plants and the empty vector-transformed plants. Moneymaker tomato plants were asymptomatic after infection with PVY. However, hpDCL2.4 plants inoculated with PVY developed symptoms, including leaf curling. Consistently, PVY CP was detected in the uninoculated symptomatic upper leaves of hpDCL2.4 plants through western blotting. Of note, PVY CP was rarely detected in other asymptomatic transgenic or wild-type plants. However, PVY was detected in the uninoculated upper leaves of all the inoculated plants using reverse transcription-polymerase chain reactions. These findings indicated that PVY systemically infected asymptomatic Moneymaker tomato plants at a low level (i.e., no detection of CP via western blotting).

**Conclusion:**

Our results indicate that the tomato cultivar Moneymaker is susceptible to PVX and shows mild mosaic symptoms, whereas it is tolerant and asymptomatic to systemic PVY infection with a low virus titer. In contrast, in hpDCL2.4 plants, PVX-induced symptoms became more severe and PVY infection caused symptoms. These results indicate that DCL2, DCL4, or both contribute to tolerance to infection with PVX and PVY. PVY CP and genomic RNA accumulated to a greater extent in DCL2.4-knockdown plants. Hence, the contribution of these DCLs to tolerance to infection with PVY is at least partly attributed to their roles in anti-viral RNA silencing, which controls the multiplication of PVY in tomato plants. The necrotic symptoms observed in the PVX-infected hpAGO2.3 plants suggest that AGO2, AGO3 or both are also distinctly involved in tolerance to infection with PVX.

## Background

The plant RNA silencing-based defense enlists a complex set of proteins to combat intracellular parasites, including viruses, retrotransposons, and other highly repetitive genome elements [1]. This defense cascade is commonly triggered by intracellularly formed double-stranded RNA (dsRNA) or partially double-stranded stem-loop RNA. These are processed by Dicer-like (DCL) nucleases into small RNAs of discrete sizes (21–25 nucleotides [nt]), referred to as small interfering RNAs (siRNAs).

siRNAs are not the end product of the cascade. Rather, they are the sequence specificity determinants of RNA-induced silencing complexes (RISC), directing Argonaute (AGO) proteins in RISC to cellular RNA or DNA complementary to the siRNAs. This process silences corresponding genes or genetic elements through targeted cleavage, repression of translation, or DNA methylation [2, 3]. In some eukaryotes, such as plants and fungi, cellular RNA-dependent RNA polymerase (RDR) acts to convert aberrant RNAs to dsRNA, leading to small RNA amplification and more intensive RNA silencing [4–6].

RNA viruses produce dsRNA as a replication intermediate, thus rendering them targets of RNA silencing [7]. DCL2 and DCL4 are largely responsible for processing viral dsRNAs into small pieces, termed virus-derived siRNAs (vsiRNAs), which can then be incorporated into RISC [8–15]. The resulting “aberrant” viral RNA cleavage products are thought to be substrates for plant RDR proteins, which subsequently generate more dsRNA [6]. The major contributors to the production of secondary vsiRNAs are RDR1, RDR2, and RDR6 [16]. Determining which AGO proteins are involved in defenses against RNA viruses has been the subject of a number of studies. In an in vitro assay, *Arabidopsis* AGO1, AGO2, AGO3, and AGO5 showed antivirus activity against a member of the genus *Tombusvirus*. In addition, specific AGOs (e.g., AGO1, AGO2, AGO3, AGO4, AGO5, and AGO9) were shown to selectively bind small RNAs derived from viroids or viruses [17–20]. These results suggest that multiple AGO proteins have the intrinsic ability to target viruses. Several AGO mutants were shown to increase susceptibility to viruses [21]; examples include AGO1 mutants and cucumber mosaic virus (CMV), turnip crinkle virus (TCV), and brome mosaic virus [15, 22–24]; AGO4 mutants and tobacco rattle virus [25, 26]; and an AGO7 mutant and TCV [15]. Among them, AGO2 appears to be a major player in RNA silencing against viruses and has been implicated in defense against CMV, TCV, tobacco rattle virus, potato virus X (PVX), turnip mosaic virus (TuMV), and tomato bushy stunt virus [20–28]. In addition, AGO5 appears to play a secondary antiviral role in the absence of AGO2 [10, 14]. Meanwhile, attenuated viruses with mutated virus silencing suppressors have also been used to identify plant factors involved in antiviral silencing, including DCL2, DCL4, AGO1, AGO2, DRB4, RDR1, RDR6, and HEN1 [6, 12, 16, 20, 27, 28]. The aforementioned studies have been mostly performed with *Arabidopsis* and *Nicotiana benthamiana*. Whether RNA silencing-related genes have similar functions and roles in crop plants is worthy of investigation.

*Potato virus X* is a type member of the genus *Potexvirus* (Alphaflexiviridae). Potato virus X (PVX) predominantly infects *Solanaceous* plants. Plants belonging to the Brassicaceae family are generally not considered to be hosts for PVX. Recent studies employing *Arabidopsis thaliana* revealed that RNA silencing is the chief determinant of the non-host immunity against PVX. Indeed, inactivation of DCLs (DCL2 and DCL4) or AGO2 enable PVX to efficiently infect *A. thaliana* plants [9, 23, 29]. Another study demonstrated that the full restriction of PVX requires AGO5 in addition to AGO2 [30].

*Potato virus Y* is a type member of the genus *Potyvirus,* family Potyviridae [31]. Potato virus Y (PVY) is a flexuous rod-shaped virus; its genome consists of a single-strand positive sense RNA (length: ~ 9.7 kb), which contains two opening reading frames (ORFs) encoding 12 proteins. One large ORF encodes a polyprotein that is cleaved into 10 functional proteins. A second small ORF, located in the P3 cistron in a different frame, encodes a polypeptide termed PIPO (Pretty Interesting Potyviridae ORF). Two proteins (P3N-PIPO and P3N-ALT) are expressed through the RNA polymerase slippage mechanism in the P3 cistron [32–37].

The tomato (*Solanum lycopersicum*) is an important vegetable crop and a model plant for the research of fruit development and plant defenses [38], including virus-derived/induced RNA silencing and plant systemic gene silencing. There are 7 DCLs (DCL1, 2a, 2b, 2c, 2d, 3 and 4), 15 AGOs (AGO1a, 1b, 2a, 2b, 3, 4a, 4b, 4c, 4d, 5, 6, 7, 10a, 10b and 15), and 6 RDRs (RDR1, 2, 3a, 3b, 6a and 6b) encoded in the tomato genome [39]. An analysis of tomato DCL1 and DCL3-silencing mutants indicated that DCL1 produces canonical miRNAs and a few 21-nt siRNAs [40], while DCL3 is involved in the biosynthesis of heterochromatic 24-nt siRNAs and long miRNAs [41]. DCL4 is required for the production of 21-nt tasiRNAs that in turn target the ARFs to alter tomato leaf development [42]. Numerous DCL2 genes (i.e., DCL2a, DCL2b, DCL2c, and DCL2d) are encoded in tomato [39]. Recently, the DCL2b-dependent miRNA pathway in tomato was shown to affect susceptibility to PVX and TMV [43]. DCL2b is also required for the biosynthesis of 22-nt small RNAs to defend against ToMV [44].

In this study, we examine the virulence of PVX and PVY in transgenic tomato plants, in which the expression of the DCL2, DCL4, AGO2, AGO3 and RDR6 genes was suppressed. Our aim in this study was to investigate whether these RNA silencing-related genes are involved in tolerance or defense against infection with these viruses in a crop plant.

## Materials and methods

### Plants and viruses

**Fig. 1:**
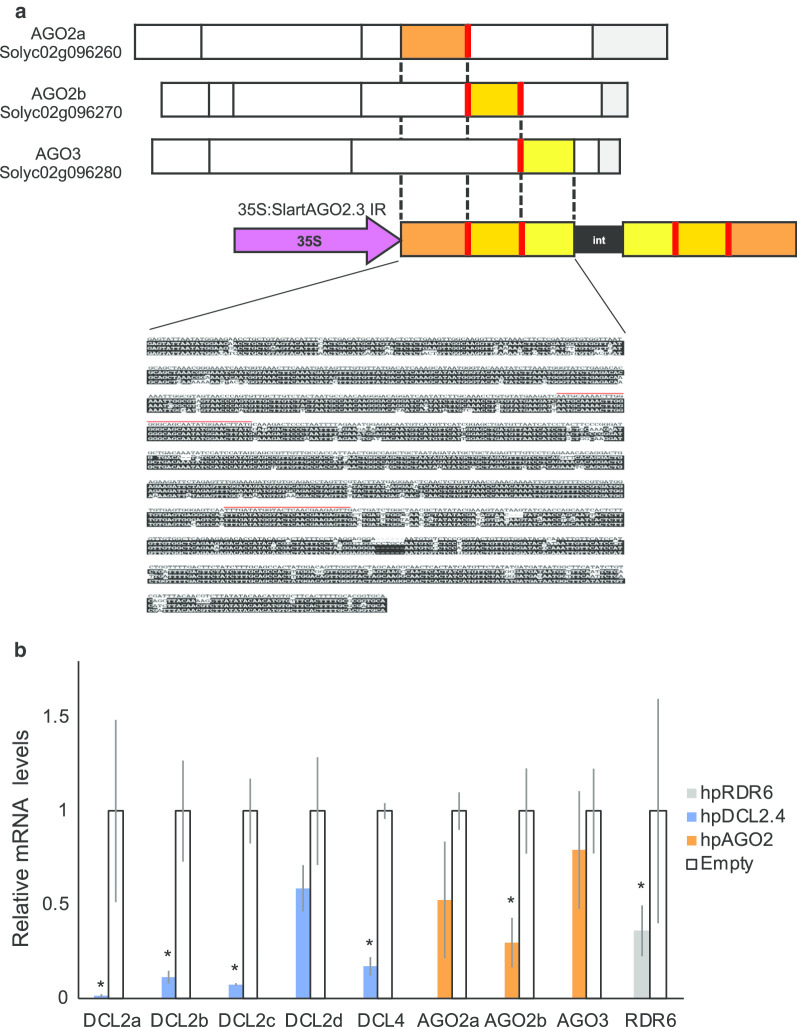
**a** Schematic diagram of the artificial chimeric genes (AGO2 and 3). Based on the alignment of the sequences of three tomato AGO genes (AGO2a, b and AGO3) registered in the database, three regions in AGO, i.e., one from AGO2a (270 bp), one from AGO2b (283 bp), and one from AGO3 (295 bp) were selected. The chimeric gene constructs were subsequently placed in head-to-head orientation across an intron sequence (‘int’ in the figure) to create an IR sequence. **b** Relative expression levels of the DCLs, AGOs, and RDR6 mRNAs in transgenic *S. lycopersicum* plants expressing dsRNAs, compared with empty vector-transformed plants. The relative levels of these mRNAs were investigated through real-time reverse transcription-polymerase chain reaction (RT-PCR) using 18S ribosome RNA as a control. Error bars represent SE. Student’s t-test was applied to analyze the data, comparing dsRNA-expressing and empty vector-transformed plants. The assay was repeated twice, and each analysis consisted of three biological replicates from three plants per treatment. Asterisks indicate a statistically significant difference in the accumulation of gene mRNA between plants with or without the expression of dsRNA (*p* < 0.05)

Wild-type tomato (*Solanum lycopersicum* cv. Moneymaker) and transgenic tomato plants created from Moneymaker were used in this study. DCL2 and DCL4- and RDR6-repressed (hpDCL2.4 and hpRDR6) tomato plants were previously created using the host RNA silencing mechanism [45, 46]. Tomato plants with AGO2a, AGO2b and AGO3 (Solyc02G069260, Solyc02G069270, and Solyc02G096280, registered in the tomato genome database at https://solgenomics.net/organism/Solanum_lycopersicum/genome) repressed (hpAGO2.3) were created in the same manner as hpDCL2.4 tomato plants [45]. The inverted repeat (IR) sequences were constructed by placing parts of AGO2, AGO3 (947 bp) in a head-to-head orientation across an intron sequence to create an IR sequence (Fig. 1a). The IR sequences were cloned into the *Bgl*II/*Kpn*I site of the binary vector pIG121-Hm [45] downstream of the CaMV-35S promoter and introduced into *Agrobacterium tumefaciens* strain EHA105 to transform the tomato plants. T3-generation plants were used in this study. In addition, tomato plants transformed with pIG121-Hm containing an empty cassette were created and used as a negative control (empty vector). PVX derived from the infectious clone pP2C2S, which was constructed with the PVX-UK3 strain [47], as well as the N [48] and O [49] strains of PVY (PVY^N^ and PVY^O^) were used for the inoculation tests in this study.

### Plant growth conditions and viral infection

Tomato plants were cultivated in a growth room at 21–23 °C with a 16-h photoperiod. PVX- and PVY-infected leaf discs (50 mg) were ground in inoculation buffer (0.1 M phosphate buffer [pH 7.0], 1% 2-mercaptoethanol). The crude sap was mechanically inoculated onto the first and second leaves of 2-week old tomato plants. At the same time, other plants were inoculated with inoculation buffer alone as a control (mock inoculation). Three plants of each transgenic and wild-type were inoculated with these viruses and analyzed.

### Reverse transcription-polymerase chain reaction (RT-PCR) and real-time PCR

PVY genomic RNA was detected using RT-PCR as follows. Tomato leaves were ground in liquid nitrogen, and total RNA was extracted with TRIzol™ Reagent (Thermo Fisher Scientific, Inc., Waltham, MA, USA). All RNA samples were treated with RNase-free DNase I (Roche Diagnostics, Basel, Switzerland). cDNA was synthesized from 1 μg of RNA extract using the modified Moloney Murine Leukemia Virus (MMLV) reverse transcriptase ReverTra Ace® (Toyobo, Osaka, Japan). PCR was performed to detect viral genomic RNA and endogenous mRNA. The mixture (25 μl) contained cDNA corresponding to 0.1 μg RNA, 0.4 μM of each of the specific primer pairs (Additional file 1: Table S1), 0.2 mM deoxyribonucleotide triphosphate (dNTPs), and 0.625 U Ex Taq™ DNA polymerase (Takara, Otsu, Siga, Japan). PCR mixtures were incubated for 2 min at 94 °C, followed by 28 cycles at 94 °C for 30 s, 60 °C for 30 s, and 72 °C for 60 s. The PCR products were fractionated through 1.2% agarose gel electrophoresis. Quantitative PCR (qPCR) was performed using the AriaMx real-time PCR system (Agilent Technologies, Santa Clara, CA, USA). The reaction mixture (25 μl) contained 0.3 mM (each) forward and reverse primers (Additional file 1: Table S1), 0.2 mM dNTPs, 0.625 U Ex Taq™ DNA polymerase (Takara), SYBR Green (1/800 dilution) (Thermo Fisher Scientific), and cDNA obtained by reverse transcribing 50 ng of total RNA. Samples were incubated for 2 min at 95 °C, followed by 39 cycles at 95 °C for 10 s, and at 58 °C and 72 °C for 20 s each.

### Northern and western blotting assays

Accumulation of the PVX and PVY CP and genomic RNA was investigated through western and northern blotting as previously described [50]. Upper uninoculated leaves from each plant at 15, 20, 25, and 30 days post-inoculation (dpi) were harvested and total RNA preparations (1–10 μg) were extracted using the TRIzol™ Reagent. After denaturing the RNA extracts by heating at 65 °C for 15 min in a solution containing RNA denaturation buffer (0.9 M disodium phosphate, 0.1 M monosodium phosphate, 37% formaldehyde, 0.05% formamide), the samples were loaded into 1.4% agarose gels containing 5% formaldehyde and 1 × 3-(N-morpholino) propanesulfonic acid buffer and run at 100 V for 30 min. Following transfer to a nylon membrane (Hybond-N; GE Healthcare, Chicago, IL, USA), hybridization with a digoxigenin-labeled probe (Roche Diagnostics) was performed to detect the 3′-terminal regions of PVX genome segments. Chemiluminescence signals with CDP-star (Millipore Sigma, St. Louis, MO, USA) were quantitatively detected using a LAS-4000 mini-imaging system (GE Healthcare).

Western blotting was conducted as previously described [51]. Total proteins were separated through electrophoresis in 12% sodium dodecyl sulfate–polyacrylamide gel electrophoresis Bis–Tris gels using Tris–glycine buffer, followed by electrotransfer onto a polyvinylidene difluoride membrane. For the detection of viral coat proteins, primary antibodies raised against PVX and PVY CP, provided by Japan Plant Protection Association, were used at a 1:1000 dilution, while alkaline phosphatase-conjugated goat antirabbit immunoglobulin G (Thermo Fisher Scientific) was used as the secondary antibody. Chemiluminescence signals were detected using the CDP-Star reagent in a LAS-4000 mini-imaging system.

## Results

### DCL2, DCL4 and AGO2 are involved in tolerance to PVX infections in tomato

**Fig. 2:**
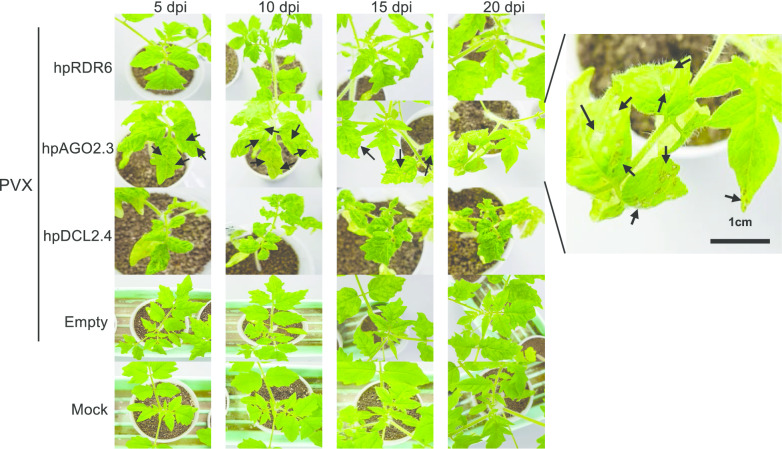
Symptoms developed in RDR6-, AGO2-, and DCL2.4-knockdown tomato plants (hpRDR6, hpAGO2.3, and hpDCL4.2) inoculated with PVX. Photographs were captured at 5, 10, 15, and 20 days-post-inoculation (dpi). The empty vector-transformed Moneymaker tomato plants were also inoculated with PVX (Empty) and buffer (Mock) as controls

**Fig. 3:**
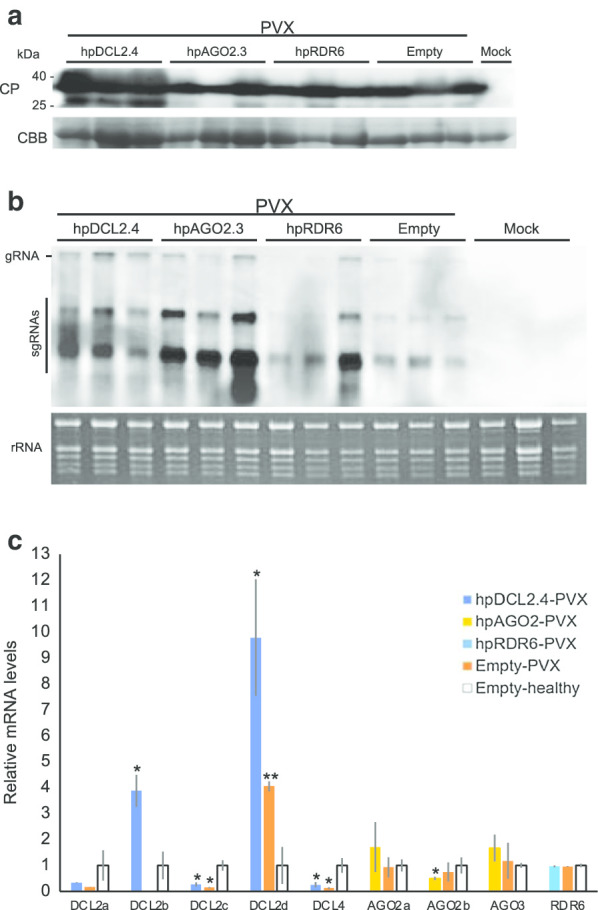
Detection of PVX CP and genomic RNA in tomato plants inoculated with PVX using western (**a**) and northern blotting (**b**). **a** Total protein samples were prepared from non-inoculated upper leaves of RDR6-, AGO2-, and DCL2.4-knockdown tomato plants (hpRDR6, hpAGO2.3, and hpDCL4.2) at 15 dpi. Samples were also prepared from the empty vector-transformed plants inoculated with PVX (Empty) and buffer (Mock) as controls. The CBB-stained gel was used as a loading control (RUBISCO protein). **b** Total RNAs extracted from hpRDR6, hpAGO2.3, hpDCL2.4, Empty, and Mock plants at 15 dpi were fractionated using an agarose gel to detect PVX genomic (gRNA) and subgenomic RNAs (sgRNAs) in non-inoculated upper leaves through northern blotting. rRNA was used as loading control. **c** RT-qPCR to compare the DCL2s, DCL4, AGO2, AGO3, and RDR6 mRNA in the upper leaves of hpDCL2.4, hpAGO2.3, hpRDR6, and empty vector-transformed plants infected with PVX (Empty-PVX) with the levels measured in healthy empty vector-transformed plants (Empty-healthy) at 15 dpi. 18S ribosome RNA was used as a control. Error bars represent SE. Student’s t-test was applied to analyze the data. Each analysis was performed with three biological replicates; data were collected from three plants of each knockdown and empty vector-transformed transgenic lines. The values with the double asterisk (**p < 0.01) and single asterisk (*p < 0.05) were statistically significant at the 1% and 5% levels, respectively, compared with those of healthy empty vector-transformed plants

We initially confirmed repression of the expression levels of DCL2, DCL4, AGO2, and RDR6 in hpDCL2.4, hpAGO2.3, and hpRDR6 plants through real-time RT-PCR. There was no significant difference in the expression levels of the DCL2d in hpDCL2.4 plants with those detected in empty-vector transgenic plants (Fig. 1b). In addition, the expression levels of DCL2a, b, c and DCL4 were also decreased to approximately 3%, 10%, 8% and 15%, respectively, in transgenic tomato plants compared with empty vector transformed plants. In hpAGO2.3 and hpRDR6 plants, the expression levels of both AGO2b and RDR6 genes were decreased to approximately 29% and 36% of the wild-type levels. In contrast, the expression levels of the AGO2a and b were not altered significantly in hpAGO2.3 plants. We inoculated these transgenic tomato plants with PVX and observed symptoms in non-inoculated upper leaves of all inoculated plants at 5 dpi (Fig. 2, Table 1). The symptoms that developed in hpDCL2.4 and hpAGO2.3 were more severe than those that developed in hpRDR6 and control transgenic plants with the empty vector. Specifically, necrotic spots began to develop in hpAGO2.3 plants on the second upper leaf from the inoculated leaf at 5 dpi, and the necrotic symptoms spread systemically at 20 dpi (Fig. 2). More severe dwarfing, as well as mosaics and leaf-malformation were observed in the hpDCL2.4 plants (Fig. 2). We investigated the accumulation of PVX CP and genomic RNA through western blotting and northern blotting (Fig. 3a, b) to determine whether the difference in symptoms was associated with the multiplication of PVX in these plants. The three transgenic tomato plants (i.e., hpDCL2.4, hpAGO2.3, hpRDR6) inoculated and those with the empty vector (Empty) all accumulated CP and the full-length genomic RNA comparably at 15 dpi (Fig. 3b). Of note, the accumulation of subgenomic RNAs differed among samples. The levels of DCLs, AGOs, and RDR6 mRNAs in transgenic lines infected with PVX were investigated by RT-qPCR (Fig. 3c). The levels of DCL2a, DCL2c, and DCL4 mRNAs in the hpDCL2.4 line infected with PVX were lower than those of the healthy empty vector-transformed plants. Unexpectedly, the mRNA levels of DCL2a, DCL2b, DCL2c, and DCL4 were also reduced in PVX-infected empty vector-transformed plants. In contrast, the mRNA levels of DCL2b and DCL2d were increased in PVX-infected hpDCL2.4 and empty vector-transformed plants. In hpAGO2.3 and hpRDR6 plants infected with PVX, the mRNA levels of AGO2, AGO3, and RDR6 were similar to those of PVX-infected and healthy empty vector-transformed plants.

**Table 1:** Reaction of transgenic plants following mechanical inoculation with PVX-UK3 and PVY^N^ strains

Host plants	Symptoms on leaves^a^			
	5 dpi	10 dpi	15 dpi	20 dpi
PVX-inoculated				
hpDCL2.4	–/M, Mal	–/M, Mal	–/M, Mal	–/M, Mal
hpRDR6	–/M	–/M	–/M	–/M
hpAGO2.3	–/M, NS	–/M, NS	–/M, NS	–/M, NS
Empty	–/M	–/M	–/M	–/M
Mock	–/–	–/–	–/–	–/–
PVY^N^-inoculated				
hpDCL2.4	–/–	–/–	–/mM, Mal	–/M, Lc
hpRDR6	–/–	–/–	–/–	–/–
hpAGO2.3	–/–	–/–	–/–	–/–
Empty	–/–	–/–	–/–	–/–
Mock	–/–	–/–	–/–	–/–

^a^Inoculated leaves/upper leaves; Lc, leaf curling; M, mosaic; mM, mild mosaic; NS, necrotic spot; Mal, malformation; Y, yellowing; –, symptomless or latent infection

### DCL2 and DCL4 are required for defense against infection with PVY

**Fig. 4:**
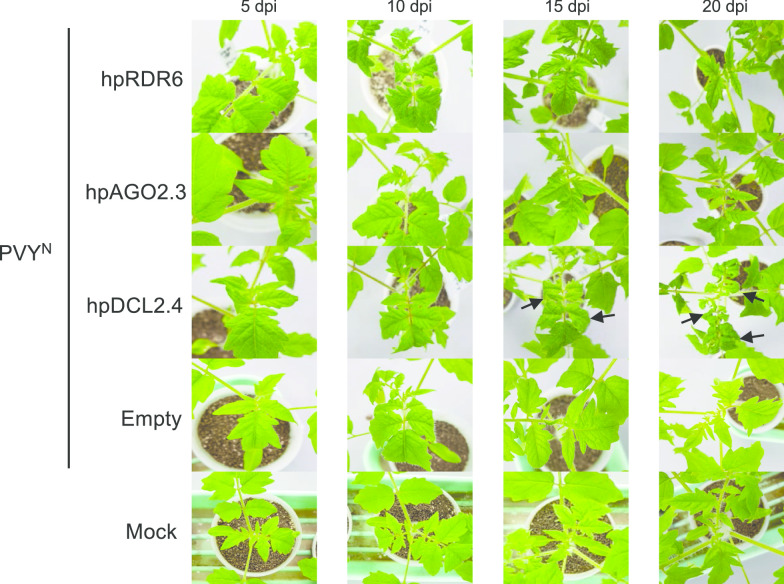
Symptoms developed in RDR6-, AGO2-, and DCL2.4-knockdown tomato plants (hpRDR6, hpAGO2.3, and hpDCL4.2) inoculated with PVY^N^. Photographs were captured at 5, 10, 15, and 20 dpi. The empty vector-transformed Moneymaker tomato plants were also inoculated with PVY^N^ (Empty) and buffer (Mock) as controls

**Fig. 5:**
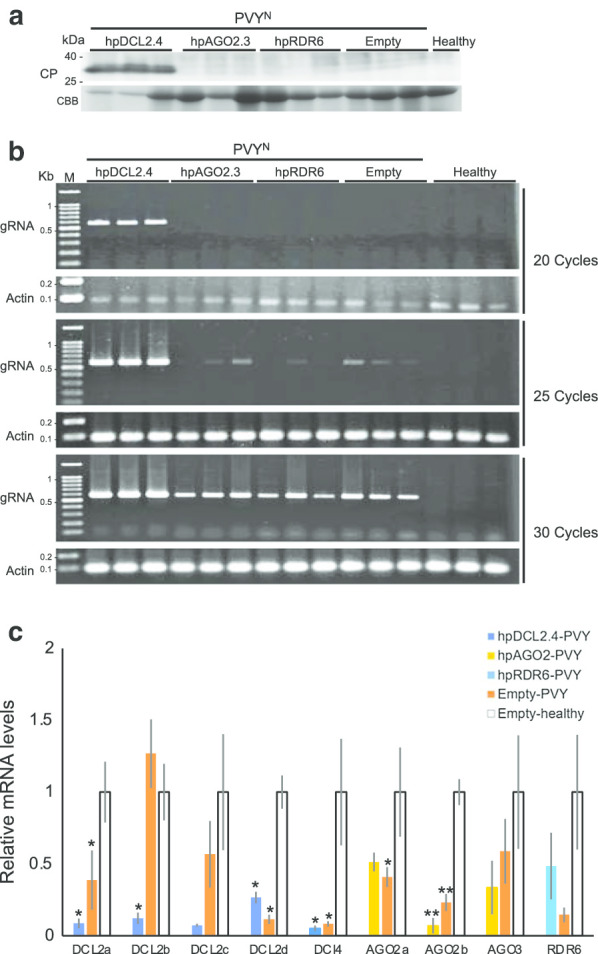
Detection of viral CP and genomic RNA (gRNA) in tomato plants inoculated with PVY^N^. **a** For the detection of CP, total protein was extracted from the upper leaves of RDR6-, AGO2-, and DCL2.4-knockdown tomato plants (hpRDR6, hpAGO2.3, and hpDCL4.2) at 15 dpi. The CBB-stained gel was used as a loading control. **b** Semi-quantitative RT-PCR for the levels of PVY^N^ genomic RNA in non-inoculated upper leaves of hpRDR6, hpAGO2.3, and hpDCL4.2 at 15 dpi. Their PCR products at 20, 25, and 30 cycles were fractionated using agarose gels. The actin gene was used as a control. **c** RT-qPCR to compare the DCL2s, DCL4, AGO2, AGO3, and RDR6 mRNA in the upper leaves of hpDCL2.4, hpAGO2.3, hpRDR6, and empty vector-transformed plants infected with PVY^N^ (Empty-PVY) with the levels detected in healthy empty vector-transformed plants (Empty-healthy) at 15 dpi. 18S ribosome RNA was used as control. Error bars represent SE. Student’s t-test was applied to analyze the data. Each analysis was performed with three biological replicates; data were collected from three plants of each knockdown and empty vector-transformed transgenic lines. The values with the double asterisk (**p < 0.01) and single asterisk (*p < 0.05) were statistically significant at the 1% and 5% levels, respectively, compared with those of healthy empty vector-transformed plants

Subsequently, we mechanically inoculated PVY^N^ into hpDCL2.4, hpAGO2.3, and hpRDR6 plants. Symptoms were only observed on the non-inoculated upper leaves of hpDCL2.4 (Fig. 4, Table 1), suggesting systemic infection of hpDCL2.4 plants with PVY^N^. PVY^N^ CP and genomic RNA were detected through western blotting and RT-PCR to confirm the systemic infection of hpDCL2.4 and to examine whether the other inoculated plants had latent systemic infections of PVY^N^. PVY CP was detected via western blotting only in DCL2.4 plants, which exclusively showed symptoms (Fig. 5a). PVY genomic RNA was detected through RT-PCR in all the inoculated plants at 15 dpi under 30 cycles of amplification. However, after 20 cycles of amplification, it was detected only in hpDCL2.4 plants (Fig. 5b). RT-qPCR detected a reduction of the mRNA levels of all tested genes in PVY^N^-infected hpDCL2.4, hpAGO2.3, and hpRDR6 plants compared with those measures in PVY^N^-infected empty vector-transformed plants. However, the mRNA levels of DCL2d, AGO2a, and RDR6 in PVY-infected empty vector-transformed plants were unexpectedly lower than those observed in PVY-infected hpDCL2.4, hpAGO2.3, and hpRDR6 plants.

**Fig. 6:**
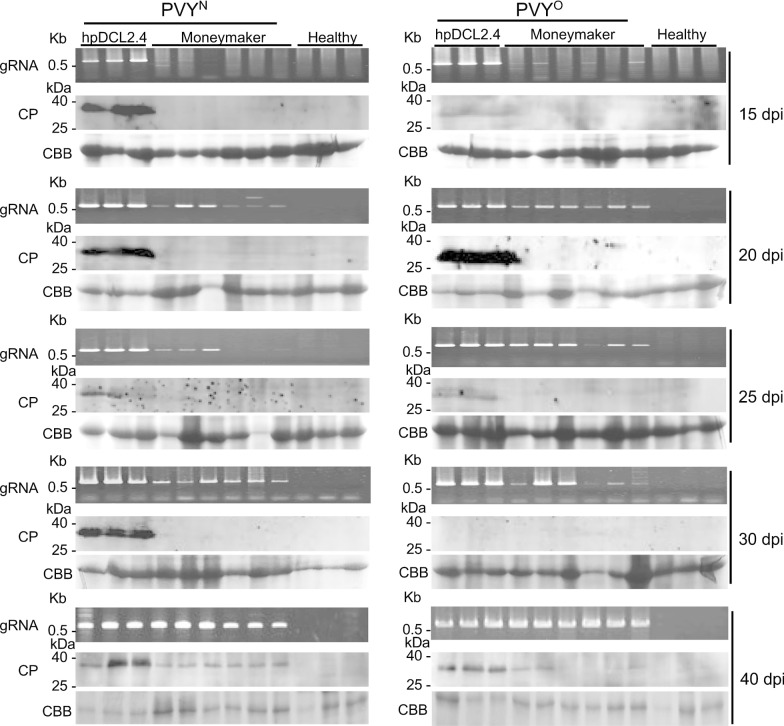
Time course analysis of PVY CP and genomic RNA (gRNA) levels in non-inoculated upper leaves of DCL2.4-knockdown tomato plants (hpDCL2.4) and the parental cultivar Moneymaker. Non-inoculated Moneymaker plants were analyzed as a control (Healthy). Total RNA and protein were extracted from the leaves of these plants at 15, 20, 25, and 30 dpi. M is the 100 bp DNA ladder. CBB-stained gels were used as a loading control (RUBISCO protein)

We further inoculated the parental cultivar and hpDCL2.4 plants with both PVY^N^ and PVY^O^, and compared their susceptibility to the viruses (Table 2). This experiment was performed to rule out the possibility that stresses during the production of transgenic plants affect their susceptibility to PVY^N^ and PVY^O^ in a strain-specific manner. As expected, hpDCL2.4 plants inoculated with both strains showed symptoms; however, none of the inoculated parental cultivar plants developed symptoms (Table 2). In addition, PVY CP was detected using western blotting only in hpDCL2.4 plants inoculated with both PVYs (Fig. 6) though PVY CP was observed in PVY-inoculated Moneymaker cultivar at 40dpi (Fig. 6). PVY genomic RNA was detected in all the samples from both PVY-inoculated plants through RT-PCR at 20 dpi and in the following days. However, at 15 dpi, PVY genomic RNA was only clearly detected in the samples obtained from hpDCL2.4 (Fig. 6). These results indicate that PVY latently and systemically infects the Moneymaker cultivar. Moreover, silencing of DCL2 and DCL4 increases the accumulation of PVY CP and genomic RNA, resulting in the development of symptoms.

**Table 2:** Reactions of transgenic plants following mechanical inoculation with PVY^N^ and PVY^O^ strains

Host plants	Symptoms on leaves^a^			
	5 dpi	10 dpi	15 dpi	20 dpi
PVY^N^-inoculated				
hpDCL2.4	–/–	–/–	–/mM, Mol	–/mM, Mol
Moneymaker	–/–	–/–	–/–	–/–
Healthy control	–/–	–/–	–/–	–/–
PVY^O^-inoculated				
hpDCL2.4	–/mM, Mol	–/mM, Mol	–/mM, Mol	–/mM, Mol
Moneymaker	–/–	–/–	–/–	–/–
Healthy control	–/–	–/–	–/–	–/–

^a^Inoculated leaves/upper leaves; Lc, leaf curling; M, mosaic; Mol, mottling; mM, mild mosaic; NS, necrotic spot; Mal, malformation; Y, yellowing; –, symptomless or latent infection

## Discussion

Silencing the DCL and AGO genes altered the reactions of a susceptible tomato plant to infection with PVX and PVY. The symptoms of PVX infection were exacerbated in DCL2, DCL4, AGO2 and AGO3-knockdown transgenic tomato plants. We observed more severe dwarfing and leaf deformation in hpDCL2.4, more severe mosaics in hpDCL2.4 and necrotic systems in hpAGO2.3 plants, compared with those in the PVX-inoculated other transgenic and wild-type plants. On the other hand, infection with PVY caused symptoms only in hpDCL2.4-knockdown plants, but not in the other transgenic or wild-type plants. RT-PCR tests showed that all PVY-inoculated plants were systemically infected with PVY, indicating that the tomato cultivar Moneymaker is susceptible to infection with PVX and PVY. These results suggest that DCL2, DCL4, AGO2 and AGO3 are involved in tolerance to infection with PVX and PVY in a susceptible tomato plant. Note that, considering the significantly increased levels of DCL2b and DCL2d mRNA in PVX-infected hpDCL2.4 plants, more severe symptoms are not necessarily caused by the downregulation of the DCL genes in plants. The higher levels of these DCL mRNAs may be attributed to the activation of their transcription [52–54], as well as the suppression of RNA silencing by PVX infection, and miR6026, which is produced by and targets DCL2s [43].

DCL2, DCL4, AGO2 and AGO3 are important factors in the RNA silencing-mediated antiviral defense [12, 15, 20, 27, 33, 55]. Therefore, it is likely that these factors contribute to tolerance through their roles in antiviral RNA silencing. This would be the case for the tolerance to infection of two strains of PVY (i.e., PVY^N^ and PVY^O^), involving DCL2 and DCL4. Western blotting was only able to detect PVY CP in inoculated hpDCL2.4 plants though it detected PVY CP at 40 dpi. RT-PCR consistently detected PVY genomic RNA in lower PCR cycle numbers and time-course experiments in samples from inoculated hpDCL2.4 plants, although all inoculated plants were systemically infected with PVY. There results indicate that the higher accumulation of PVY observed in hpDCL2.4 plants can probably be attributed to the reduced activity of RNA silencing against PVY. DCL proteins possess RNase III activity to generate small RNAs, such as siRNAs and microRNAs (miRNAs) [55], and silencing or mutations in DCLs would affect siRNA biogenesis. We recently showed a defect in the biogenesis of siRNAs, especially 22 nt siRNAs, derived from viroid RNA in hpDCL2.4 plants infected with the potato spindle tuber viroid [44]. Studies have reported the increased accumulation of CMV genomic RNAs [12] and increased susceptibility to PVX and the TuMV helper component-protease mutant in *Arabidopsis* DCL double (DCL2 and 4) and triple mutants (DCL2, 3, and 4) [56]. Increased accumulation of TuMV and CMV genomic RNAs was observed in DCL2- and DCL4-knockdown *N. benthamiana* [56]. Silencing of DCL4 facilitates the systemic movement of *Zucchini yellow mosaic virus* in *N. benthamiana* [54]. These studies indicate that DCL2 and DCL4 restrict the multiplication of viruses in susceptible plants. In this study, tomato DCL2 and DCL4 also did not completely prevent infection with PVY. However, they efficiently restricted infection to reduce the occurrence of symptoms in a susceptible cultivar.

Tolerance to infection with PVX does not appear to be attributed to the roles of DCL2, DCL4, and AGO2 in antiviral RNA silencing. This conclusion was based on the absence of obvious difference in CP and genomic RNA levels among hpDCL2.4, hpAGO2.3 transgenic and wild-type tomato plants (Fig. 4a). PVX has a relatively weak RNA silencing suppressor, triple gene block protein 1 (TGBp1) [57], and may have ability to escape or survive under active conditions of antiviral RNA silencing [58]. This ability may partly explain the absence of an obvious increase in PVX accumulation in DCL2.4 and AGO2 plants (Fig. 4b). RNA silencing is one of the major antiviral defense mechanisms involved in the regulation of numerous endogenous genes via siRNAs and miRNAs [59]. Thus, symptom exacerbations may be attributed to differences in the expression of endogenous genes via the knockdown of DCL2, DCL4, AGO2 and AGO3. We recently showed that symptom exacerbations in hpDCL2.4 plants infected with potato spindle tuber viroid could be attributed to the increased expression of miR398a-3p, which increased the production of reactive oxygen species [44].

Interactions between PVX and AGO2 have previously been studied. Similar symptom exacerbations, including systemic necrosis, have been observed in the AGO2-knockout *N. benthamiana* using CRISPR/Cas9 [60]. The *Arabidopsis* Col-0 plant is a non-host for PVX; however, PVX becomes capable of multiplication in inoculated leaves following mutation of AGO2 [23]. P25, also known as triple gene block protein 1 (TGBp1), suppresses RNA silencing [61, 62], which can be partly explained by P25 binding to and directing the degradation of AGO1 via the 26S proteasome [63]. Studies have shown that P25 has an affinity for AGO2 [23, 63]. On the other hand, necrosis or cell death associated with infection with PVX or closely related viruses that belong to the genus *Potexvirus* has been reported. Infection with PVX triggers hypersensitive cell death responses in potato plants carrying the Nb gene, and P25 is the elicitor of these responses [64]. TGBp3 induces the unfolded protein response during infection with PVX. This effect is important in the regulation of cellular cytotoxicity that could otherwise lead to cell death if the viral proteins reach high levels in the ER [65, 66]. An isolate of the plantago asiatica mosaic virus causes systemic necrosis in *N. benthamiana*, and its RNA-dependent RNA polymerase is a virulence determinant for necrotic symptoms [67]. Additionally, an isolate of the PVX-OS strain induced systemic necrotic mosaic in *Nicotiana* spp, and the 1422 amino acid C-terminal of its RNA-dependent RNA polymerase is a determinant of systemic necrotic mosaic symptoms [68]. Co-infection with PVX and PVY causes systemic necrosis in tobacco plants, and PVX P25 and PVY helper component-protease have been identified as determinants for necrotic symptoms [69]. Recently, systemic necrosis was correlated with an enhanced expression of lipoxygenase activity in PVX and PVY co-infected plants [70]. Lipoxygenase acts on polyunsaturated fatty acid substrates in the first step of the biosynthetic pathway of jasmonic acid, a hormone involved in the execution of hypersensitive response cell death in tobacco [71]. Downregulation of double-stranded-RNA-Binding Protein (DRB2) by VIGS is able to reduce PVX-triggered systemic necrosis in ago2 mutant *N. benthamiana* [72].

These previous studies may help reveal the mechanism through which AGO2-knockdown alters the expression of endogenous genes and which host and viral genes involved in the development of necrotic symptoms during infection with PVX.

## Conclusion

In this study, we observed an increased accumulation of PVY CP and genomic RNA with symptoms in hpDCL2.4 plants, suggesting that DCL2 and DCL4 are involved in anti-PVY defense in tomato plants via the RNA silencing mechanism. Although all the PVX-inoculated transgenic plants comparably accumulated CP and genomic RNA, more severe and additional necrotic symptoms were observed in hpDCL2.4 and hpAGO2.3 plants. Based on the present findings, DCL2, DCL4 and AGO2 are involved in tolerance to infection with PVX and PVY in tomato.

## Supplementary information

**Additional file 1: Table S1**. Primers used for cDNA amplification and qRT-PCR

## Data Availability

The datasets used during the current study are available from the corresponding author with an appropriate request.

## Abbreviations

AGO: Argonaute
CMV: cucumber mosaic virus
CP: coat protein
CRISPR: clustered regularly interspaced short palindromic repeats
DCL: Dicer-like
DIG: digoxigenin
dpi: days post inoculation
DRB: double-stranded RNA binding
ER: endoplasmic reticulum
HEN: HUA ENHANCER
HR: hypersensitive response
IR: inverted repeat
miRNA: microRNA
ORF: opening reading frames
PIPO: Pretty Interesting Potyviridae ORF
PVX: potato virus X
PVY: potato virus Y
RISC: RNA-induced silencing complexes
RT-PCR: reverse transcription-polymerase chain reaction
siRNA: small interfering RNA
TCV: turnip crinkle virus
TuMV: turnip mosaic virus
TGB: triple gene block
vsiRNA: virus-derived siRNA
