# Supplementary material for: RNA silencing-related genes contribute to tolerance of infection with potato virus X and Y in a susceptible tomato plant
Source: Virol J. 2020 Oct 8;17:149. doi: 10.1186/s12985-020-01414-x (PMC7542965; doi:10.1186/s12985-020-01414-x)
Supplement: Supplementary file 1 — Additional file 1: Table S1. Primers used for cDNA amplification and qRT-PCR [file 12985_2020_1414_MOESM1_ESM.pdf]

Table S1. Primers used for cDNA amplification and qRT-PCR

Primer name	Nucleotide sequence (5 ′ → 3′)
SlDCL2a-F	GCTCGGGACGAGACAAAATA
SlDCL2a-R	GGAAGGCACATCCAAACAGT
SlDCL2b-F	GAATGTCCATCTAATGCTACTGAAC
SlDCL2b-R	AACGTGTCGTCTCACAAAGC
SlDCL2c-F	TTGTTTGTGGAAACCAGCTAG
SlDCL2c-R	TCTTTCCAGAACCAGTCTCCA
SlDCL2d-F	CAGGGCGTATTACTACGTTCAA
SlDCL2d-R	CAAGCCCTCTTCAAGAATCG
SlDCL4-F	GGAAGAGGTCGTAGAGCGATT
SlDCL4-R	CACACCAATAACAGGCCACA
SlAGO2a-F	TTCTTCCTCCCCCTGATTTG
SlAGO2a-R	CTTGCCTTCCACCACAGATT
SlAGO2b-F	ACGCAGACCTTGTTGCTTAC
SlAGO2b-R	TCTTTGTTCAAATGAGGCAG
SlAGO3-F	CTAGGCGGTCAAAATCCACC
SlAGO3-R	GCCCATCGTTGAAGTGCCTT
SlRDR6-F	GCGGCTATAATGTTGAGTGCAGGG
SlRDR6-R	GTCTTATTCCTGAGGTCGCCAAGC
SlActin-F	TGGGATGATATGGAGAAGATATGG
SlActin-R	GGCTTCAGTTAGGAGGACAGGA
Ps18SrRNA-F	CGTTCTTAGTTGGTGGAGCGAT
Ps18SrRNA-R	CCATAGTCCCTCTAAGAAGCTG
PVYCP-F	CGTCCAAAATGAGAATGCC
PVYCP-R	TCTTGTGTACTGATGCCAC

Primers used for RT-PCR and RT-qPCR were listed. It includes specific primers for PC R to amplify cDNAs of tomato DCLs, AGOs, RDR6 and PVY genome, and those for ref erence genes, tomato actin and 18S ribosomal RNA in RT-qPCR.
